# Using *Pox-Neuro* (*Poxn*) Mutants in *Drosophila* Gustation Research: A Double-Edged Sword

**DOI:** 10.3389/fncel.2018.00382

**Published:** 2018-10-24

**Authors:** Yu-Chieh David Chen, Scarlet Jinhong Park, William W. Ja, Anupama Dahanukar

**Affiliations:** ^1^Interdepartmental Neuroscience Program, University of California, Riverside, Riverside, CA, United States; ^2^Department of Neuroscience, The Scripps Research Institute, Jupiter, FL, United States; ^3^Department of Molecular, Cell and Systems Biology, University of California, Riverside, Riverside, CA, United States

**Keywords:** *Drosophila*, *Pox-neuro*, pharyngeal taste, gustation, feeding behavior, taste-blind

## Abstract

In *Drosophila*, *Pox-neuro* (*Poxn*) is a member of the Paired box (Pax) gene family that encodes transcription factors with characteristic paired DNA-binding domains. During embryonic development, *Poxn* is expressed in sensory organ precursor (SOP) cells of poly-innervated external sensory (p-es) organs and is important for specifying p-es organ identity (chemosensory) as opposed to mono-innervated external sensory (m-es) organs (mechanosensory). In *Poxn* mutants, there is a transformation of chemosensory bristles into mechanosensory bristles. As a result, these mutants have often been considered to be entirely taste-blind, and researchers have used them in this capacity to investigate physiological and behavioral functions that act in a taste-independent manner. However, recent studies show that only external taste bristles are transformed in *Poxn* mutants whereas all internal pharyngeal taste neurons remain intact, raising concerns about interpretations of experimental results using *Poxn* mutants as taste-blind flies. In this review, we summarize the value of *Poxn* mutants in advancing our knowledge of taste-enriched genes and feeding behaviors, and encourage revisiting some of the conclusions about taste-independent nutrient-sensing mechanisms derived from these mutants. Lastly, we highlight that *Poxn* mutant flies remain a valuable tool for probing the function of the relatively understudied pharyngeal taste neurons in sensing meal properties and regulating feeding behaviors.

## Introduction

Taste is essential for insects to evaluate the palatability and nutritional content of food sources and to make important decisions on feeding, mating, and egg laying (Dethier, 1976; Scott, 2018). An understanding of the insect taste system may lead to the development of new strategies to control insect feeding behaviors, which constitute a significant economic and health burden each year. The vinegar fly, *Drosophila melanogaster*, has been a highly tractable model organism to explore the neurobiology of insect taste. With the powerful molecular genetic tools and robust behavioral assays in this model, scientists have explored how taste information is recognized and processed to control feeding behaviors.

In *Drosophila*, there are two major types of external sensory bristles distinguished broadly as mono- or poly-innervated based on the number of neurons that are housed within. Mono-innervated external sensory (m-es) bristles, such as mechanosensory bristles, are distributed all over the body. Each is innervated by a single mechanosensory neuron, which extends its dendrite to the base of the shaft and detects deflection of the hair (Falk et al., 1976). Poly-innervated external sensory (p-es) bristles, such as taste bristles, are distributed in various parts of the body, including the labellum, distal segments of the legs, wing margins, and the ovipositor (Stocker, 1994; Liman et al., 2014; Freeman and Dahanukar, 2015). Within the taste bristles, there are multiple taste neurons (usually 2–4 in the labellar taste bristles) that extend their dendrites up to the tip of the hair shaft, close to a single pore through which tastants can enter the sensillum lymph. During development, sensory mother cells of different lineages generate different type of sensory organs, specified by sets of transcription factors (Ghysen and Dambly-Chaudiere, 2000). One such factor is Pox-neuro (Poxn), which is a transcription factor with a paired DNA-binding domain. During neurogenesis, Poxn is expressed in sensory mother cells that eventually give rise to p-es organs of the peripheral nervous system (Dambly-Chaudiere et al., 1992). In *Poxn* mutants, all external chemosensory bristles are transformed into mechanosensory bristles (Dambly-Chaudiere et al., 1992; Awasaki and Kimura, 1997), offering a model with numerous possible uses in gustatory research.

The identification of candidate taste receptor genes in early 2000 was a major breakthrough in understanding the molecular and cellular basis of insect gustation (Clyne et al., 2000). By scanning for predicted structural properties of encoded proteins rather than specific DNA sequences, John Carlson’s group at Yale University identified a transmembrane receptor family that shared no sequence similarity to any known proteins. Many members of this family were expressed in a major gustatory organ, the labellum, which informed its naming as the *Gustatory receptor* (*Gr*) gene family. Importantly, *Poxn* mutants were used to support taste-specific or taste-enriched expression of selected *Gr* genes. A comparison of *Gr* expression between wild-type and *Poxn* mutant flies uncovered that 18 of 19 *Gr* transcripts were not detected in mutant labella (Clyne et al., 2000). This study was the first to demonstrate the utility of *Poxn* mutants for identifying the *Gr* gene family, which was quickly followed by further characterization of additional *Gr* members and analysis of their expression with transgenic reporters (Scott et al., 2001).

Subsequently, *Poxn* mutants and related genetic tools have been widely used for gustation research in *Drosophila*. In this mini-review, we examine how *Poxn* manipulations were used to reveal additional taste sensillum-enriched genes and discuss examples of how *Poxn* mutants have been utilized in behavioral research to dissect the involvement of gustatory sensory inputs as well as to identify taste-independent nutrient-sensing mechanisms. Finally, we highlight recent studies confirming that internal pharyngeal taste neurons remain intact in *Poxn* mutants (LeDue et al., 2015; Chen and Dahanukar, 2017), indicating that these mutants are not taste-blind. We suggest that the importance of pharyngeal input in driving feeding behaviors should be considered and explored further, and that some conclusions of previous studies should be reevaluated in light of these recent findings.

## *Poxn* Mutants as a Valuable Tool for Identifying Taste Sensillum-Enriched Genes

The absence of gustatory bristles in *Poxn* mutants enabled the identification of taste-related genes, whose expression was expected to be down-regulated in the mutants as compared to control flies. This rationale was validated through numerous studies and confirmed the value of *Poxn* as a tool for such molecular discoveries. For example, by using RT-PCR or microarray analysis of cDNA from taste organs in wild-type and *Poxn* mutants, many genes that were enriched in wild-type relative to *Poxn* mutants were identified as chemosensory receptor genes, including the *Gr* (Clyne et al., 2000; Ueno et al., 2001; Moon et al., 2009), *ionotropic receptor* (*Ir*) (Koh et al., 2014), and *pickpocket* (*Ppk*) (Cameron et al., 2010; Lu et al., 2012, 2014) gene families. Similar strategies were used to reveal expression of other genes in external taste organs, including those that encode odorant-binding proteins (Koganezawa and Shimada, 2002; Jeong et al., 2013) and the adipokinetic hormone receptor (Bharucha et al., 2008), which led to the characterization of their roles in taste detection and feeding behavior.

More recently, molecular genetic tools derived from the *Poxn* locus were used to alter sensory bristles in a taste organ-specific manner. Taking advantage of the *GAL4/UAS* system (Brand and Perrimon, 1993) to induce tissue-specific RNAi, Raad et al. (2016) found that silencing of *Poxn* in wings caused all taste bristles in the anterior wing margin to be transformed into mechanosensory bristles, leaving those in other taste organs intact. Such targeted silencing of *Poxn* in specific tissues could be of value for dissecting roles of different taste organs in chemosensory behaviors. In addition, several *Poxn-GAL4* transgenes synthesized with various *Poxn* enhancers are available and can be used to gain genetic access to the vast majority of taste neurons (Boll and Noll, 2002). Both *GAL4* and *UAS* transgenic reagents of *Poxn* have been used to label or to knock down genes of interest in taste neurons. More recently, *Poxn-GAL80* has also been generated for blocking GAL4 activity in most if not all taste neurons (Steck et al., 2018). Together, the *Poxn* molecular genetic toolkit (Table 1) has the necessary components for executing intersectional strategies to broadly manipulate taste hairs.

**Table 1:** The *Poxn* toolkit.

*Poxn* tools	Purpose	Reference
*Poxn-GAL4*	Genetic access to most taste neurons via the *GAL4/UAS* system	Boll and Noll, 2002; Bhandari et al., 2006; Mellert et al., 2010; Wang et al., 2011; Liu et al., 2012; Starostina et al., 2012; Park et al., 2013; Lu et al., 2014; Vijayan et al., 2014; Clowney et al., 2015; Yilmazer et al., 2016; Chowdhury et al., 2017; Sovik et al., 2017; Kojima et al., 2018; Steck et al., 2018
*Poxn-GAL80*	Block activity of the *GAL4/UAS* system in most taste neurons	Steck et al., 2018
*UAS-Poxn RNAi*	Tissue-specific *Poxn* knockdown	Raad et al., 2016; Houot et al., 2017
*Poxn-CD8::GFP*	GFP expression under direct control of *Poxn* enhancer	Minocha et al., 2017
Anti-Poxn antibody	Poxn expression	Diaper et al., 2013

## *Poxn* Mutants are Not Taste-Blind

The *Poxn* mutant has been widely used to investigate the importance of taste sensory input in driving behaviors of interest (Table 2). The underlying assumption for many of these studies was the taste-blind feature of *Poxn* mutant flies. In instances where *Poxn* mutants exhibited behaviors similar to those of wild-type counterparts, the palpable conclusion was that the observed behaviors were generated by taste-independent mechanisms. However, several studies provided hints that internal pharyngeal taste organs are intact in *Poxn* mutants (Galindo and Smith, 2001; LeDue et al., 2015; Chen and Dahanukar, 2017). Analysis of odorant binding protein (OBP) expression revealed that *Poxn* mutants lose expression of external gustatory-specific OBPs but not of ones in the pharynx, such as OBP56b (Galindo and Smith, 2001). This study, published in 2001, was the first to posit a specific requirement for *Poxn* in cell fate determination of external but not internal taste organs. It was not until much later that a functional demonstration followed, in a study that found intact pharyngeal *Gr43a* taste neurons in *Poxn* mutants and proved their requirement for sugar selection and sustained consumption (LeDue et al., 2015).

**Table 2:** *Poxn* mutants for behavioral research.

Behaviors	Phenotype	Reference
Feeding	Nutrient sensing	Dus et al., 2011, 2013; Abu et al., 2018
	Sugar	Usui-Aoki et al., 2005; Sun et al., 2014; LeDue et al., 2015; Liu et al., 2015; Murata et al., 2017
	Bitter	Mitri et al., 2009; Chen and Dahanukar, 2017
	Salt	Kojima et al., 2018
	pH	Deshpande et al., 2015
	Yeast	Steck et al., 2018
	Water	Chen et al., 2010
	Ethanol	Devineni and Heberlein, 2009
	Fatty acid	Masek and Keene, 2013
Social	Aggregation pheromone detection	Lin et al., 2015
	Social interaction	Schneider et al., 2012; Schneider and Levine, 2014
Reproductive	Oviposition	Joseph et al., 2009; Joseph and Heberlein, 2012; Hussain et al., 2016; Verschut et al., 2017
	Courtship	Boll and Noll, 2002; Krstic et al., 2009
Others	Grooming	Yanagawa et al., 2014, 2018
	Positional preference	Joseph et al., 2009; Joseph and Heberlein, 2012
	Starvation-induced hyperactivity	Yang et al., 2015

These results set the stage for a comprehensive study of chemosensory receptor expression in the pharynx, which showed that pharyngeal taste neurons and their central projections in the taste center, the subesophageal zone, are intact in *Poxn* mutants (Chen and Dahanukar, 2017). Thus, although *Poxn* mutants have lost all external taste bristles, they are not rendered taste-blind by virtue of taste sensory neurons preserved in the pharynx–the role of pharyngeal taste in driving *Poxn* behaviors that were thought to be taste-independent should therefore be evaluated. For example, previous studies suggested that, in addition to sweetness, the caloric content of sugar can also drive food preference. Taste-independent detection of the caloric content of sugar was evaluated using *Poxn* mutants, which were insensitive to the taste of sugar in proboscis extension assays but exhibited preference for the nutritive sugar in feeding assays (Dus et al., 2011, 2013). Given that pharyngeal *Gr43a* taste neurons are still functional and drive selection of both nutritive and non-nutritive sugars in *Poxn* mutants (LeDue et al., 2015), the possibility of their functional interactions with neurons identified as having internal nutrient-sensing capabilities, such as SLC5A11- (Dus et al., 2011, 2013) or DH44-neurons (Dus et al., 2015), cannot be ruled out. In addition, a recent study showed that another group of pharyngeal taste neurons expressing *Ir60b* responds strongly to sucrose but weakly to glucose (Joseph et al., 2017). Interestingly, *Ir60b* mutants have specific defects in sensing sucrose but not in detecting other nutritive or non-nutritive sugars, suggesting that there are distinct pharyngeal sugar-sensing mechanisms that allow discrimination between various sugars. It will be of interest to evaluate functional intersections of pharyngeal taste and nutrient sensing in the future.

Nevertheless, *Poxn* mutants present a useful vehicle for dissecting the role of external taste input in many behaviors of interests (Table 2). In many behavioral assays, *Poxn* mutants have shown a degree of deficit as compared to wild-type controls, indicating contributions of information from external taste organs in oviposition site selection (Joseph et al., 2009; Hussain et al., 2016), the effect of pheromones on life span and physiology (Gendron et al., 2014), trehalose consumption (Usui-Aoki et al., 2005), and the effect of food pH on palatability (Deshpande et al., 2015).

## Pharyngeal Taste Presents a Missing Link Between Taste Input and Feeding Behavioral Output

Given the anatomical location of pharyngeal taste organs, it has long been assumed that they act as gatekeepers for monitoring food quality and controlling ingestion, but there is little direct knowledge of the functional roles of sensory neurons that reside within. *Poxn* mutants offer a minimal taste model for probing the roles of pharyngeal taste neurons in feeding behaviors. In the context of *Poxn* mutants, pharyngeal sensitivity to tastants other than sweet compounds has not been explored in depth, and there are recent studies hinting at the function of pharyngeal taste neurons in detecting bacterial lipopolysaccharides (Soldano et al., 2016) and high concentrations of salt (NaCl) (Kim et al., 2017). A comprehensive examination of pharyngeal taste receptivity has not yet been done, but pharyngeal expression of chemosensory receptors involved in sensing water (Cameron et al., 2010), bitter (Weiss et al., 2011), salt (Zhang et al., 2013), and electrophiles (Kang et al., 2010) implies that the potential for detecting other categories of tastants exists. Indeed, *Poxn* mutants are capable of selecting appetitive tastants such as sugars and amino acids, and rejecting aversive tastants such as bitter compounds, high salt concentration, and very low pH, suggesting that pharyngeal taste organs pose an important link between taste sensory input and feeding behavioral output. With the pharyngeal receptor-to-neuron maps established recently (Chen and Dahanukar, 2017), it is now possible to use genetic dissection strategies to interrogate the function of different neuronal subsets in driving behavioral responses to various tastants. We expect that such experiments will be of value, not only to demonstrate the contributions of pharyngeal taste neurons in controlling food intake, but also to probe the sensory functions of the many remaining orphan neurons.

## The Function of *Poxn* in the Developing Nervous System

*Poxn* mutants have been described as having defects in p-es organs but not in m-es organs. The mechanisms underlying the specificity of this defect are not yet clear, but it is conceivable that internal pharyngeal taste organs rely on other transcription factors and signaling networks. In fact, there is a difference in developmental timing between adult pharyngeal taste neurons, which are born during embryogenesis and persist through metamorphosis (Gendre et al., 2004), and external taste neurons that originate only during metamorphosis. Notably, many olfactory sensilla in the antennae and maxillary palps also house multiple olfactory receptor neurons (1–4 ORNs). However, olfactory sensilla do not appear to be affected in *Poxn* mutants, since ORN projections remain intact in the antennal lobes (Chen and Dahanukar, 2017). Thus, *Poxn* mutants have specific defects in gustatory but not olfactory bristles.

In addition to the peripheral nervous system, *Poxn* is also expressed in various postmitotic neurons in the developing brain, including a protocerebral dorsal cluster and a deutocerebral ventral cluster (Minocha et al., 2017). The former is crucial for connections of the bulb with the ellipsoid body, while the latter is important for connections between the antennal lobe and lateral horn. In *Poxn* mutants, the *Poxn*-expressing brain neurons cannot establish proper connections with their targets. The behavioral consequences of the central nervous system defects are not clear and await further characterization. Although the wiring defects were observed in *Poxn* mutants homozygous for the *ΔM22-B5* allele, created by an imprecise excision spanning over 17 kb that removes part of the *Poxn* gene and promoter as well as an adjacent gene encoding a sugar transporter homolog, CG8249 (Boll and Noll, 2002), a recent study pinpoints a 1442 kb upstream fragment as an important enhancer for brain function (Minocha et al., 2017). In addition to the defects in the central nervous system, mutants homozygous for the *ΔM22-B5* allele also have defects in leg/antenna segmentation, male courtship, male fertility, and flight (Boll and Noll, 2002). Although defined enhancer regions have been implicated for specific functions (Boll and Noll, 2002), little is known about involvement of the adjacent gene that is removed in the *ΔM22-B5* allele in fly behavior. An ethyl methanesulfonate (EMS)-generated allele, *Poxn^70^*, has been reported to be an amorphic allele (Awasaki and Kimura, 1997) with adjacent genes likely intact. Thus, the transheterozygous allelic combination of *Poxn^Δ*M22*-*B5*^*/*Poxn^*70*^* in recent studies might circumvent some of the defects described in flies homozygous for the *ΔM22-B5* allele (LeDue et al., 2015; Chen and Dahanukar, 2017), although this remains unconfirmed.

## Considerations in Using *Poxn* Mutants for Gustation Research

The proboscis extension reflex (PER) assay has been tremendously valuable as a measure of taste behavior response (Shiraiwa and Carlson, 2007). However, while *Poxn* mutants lack functional external taste bristles and are insensitive to labellar or tarsal stimulations with appetitive sugar solutions, they are indeed able to identify and consume food, enabling the use of the many food intake assays available for assessing contributions of pharyngeal taste neurons to feeding behaviors. Given that *Poxn* mutants are defective in external taste sensing, care must be taken in selecting appropriate assays for quantifying food intake. An increasing number of assays have been developed for measuring food intake in *Drosophila*, including the quantification of food labeled with radiotracers or colorimetric dyes, or direct monitoring of consumed volume of liquid diet (solutions of yeast or sugar) in the Capillary Feeder (CAFE) assay (Deshpande et al., 2014). *Poxn* mutants have been shown to ingest food as either liquid (i.e., in the CAFE assay) (Devineni and Heberlein, 2009) or solid (i.e., radiolabeling or colorimetric dyes in agar-based medium) (Deshpande et al., 2015; LeDue et al., 2015; Chen and Dahanukar, 2017). However, a recent report showed that *Poxn* mutant flies have difficulty in finding food sources with increased distance between them in binary choice assays (Abu et al., 2018), suggesting a context-dependent foraging deficiency in *Poxn* flies. Recently developed tools such as FlyPAD, which can be used for high resolution quantification of contacts with food, showed normal yeast feeding behavior in *Poxn* mutants (Steck et al., 2018), and thus offer alternatives for testing *Poxn* mutants.

In addition to the effects of different *Poxn* mutant alleles on the development of the central nervous system, another precaution in using *Poxn* mutants in gustatory research is that there has been no assessment of whether the supernumerary mechanosensory bristles have any function in mechanosensing and thus impart hypersensitivity to mechanical stimuli. Indeed, recent studies have identified at least two different neuronal populations that mediate feeding preference on the basis of texture. One class is the mechanosensory neurons in labellar taste sensilla, which express a mechanosensory receptor, NOMPC (Sanchez-Alcaniz et al., 2017). The second is multidendritic neurons in the labellum (md-L), expressing the transmembrane channel-like (TMC) protein (Zhang et al., 2016). It is not clear how these two mechanosensing mechanisms interact, but the contribution of mechanosensation in feeding behaviors cannot be ignored. Ensuring that all genetic manipulations and comparisons use the same *Poxn* mutant background will help minimize or rule out hypersensitivity in mechanosensing, as well as other potential defects, as confounds in interpreting results.

## Conclusion and Perspective

Over the last couple of decades, the *Poxn* mutation, which specifically affects the developmental fate of gustatory bristles, has presented unique opportunities for investigating molecular and cellular principles of taste system function. The *Poxn* mutant has been subjected to a diverse range of approaches, spanning differential gene analysis for identifying taste-related genes, to behavioral analysis for identifying the contribution of specific gustatory inputs. Importantly, recent studies have shown that all internal pharyngeal taste organs remain intact in *Poxn* mutants, which brings immediate attention to the research community that *Poxn* mutants are not taste-blind and warrants revisiting taste-independent nutrient-sensing mechanisms established through their use. Instead, the *Poxn* mutant provides a model with a minimal pharyngeal taste system with which to dissect the function of pharyngeal taste neurons. Combined with the genetic toolkit derived from the recently described map of pharyngeal taste neurons, we now have the means to evaluate the sensory function of a taste organ that has often been overlooked while interpreting results of feeding behavior experiments.

## Author Contributions

Y-CC, WJ, and AD conceptualized the study. Y-CC wrote the original draft. Y-CC, SP, WJ, and AD wrote, reviewed, and edited the manuscript. WJ and AD supervised the study. WJ and AD acquired funding.

## Conflict of Interest Statement

The authors declare that the research was conducted in the absence of any commercial or financial relationships that could be construed as a potential conflict of interest.

## References

[B1] Abu F. Wang J. G. Oh Y. Deng J. Neubert T. A. Suh G. S. B. (2018). Communicating the nutritional value of sugar in *Drosophila*. *Proc. Natl. Acad. Sci. U.S.A.* 115 E2829–E2838. 10.1073/pnas.1719827115 29507251 PMC5866586

[B2] Awasaki T. Kimura K. (1997). pox-neuro is required for development of chemosensory bristles in *Drosophila*. *J. Neurobiol.* 32 707–721. 10.1002/(SICI)1097-4695(19970620)32:7<707::AID-NEU6>3.0.CO;2-8 9183748

[B3] Bhandari P. Gargano J. W. Goddeeris M. M. Grotewiel M. S. (2006). Behavioral responses to odorants in drosophila require nervous system expression of the beta integrin gene myospheroid. *Chem. Senses* 31 627–639. 10.1093/chemse/bjl002 16763085

[B4] Bharucha K. N. Tarr P. Zipursky S. L. (2008). A glucagon-like endocrine pathway in *Drosophila* modulates both lipid and carbohydrate homeostasis. *J. Exp. Biol.* 211 3103–3110. 10.1242/jeb.016451 18805809 PMC2714167

[B5] Boll W. Noll M. (2002). The Drosophila pox neuro gene: control of male courtship behavior and fertility as revealed by a complete dissection of all enhancers. *Development* 129 5667–5681. 10.1242/dev.00157 12421707

[B6] Brand A. H. Perrimon N. (1993). Targeted gene expression as a means of altering cell fates and generating dominant phenotypes. *Development* 118 401–415. 8223268 10.1242/dev.118.2.401

[B7] Cameron P. Hiroi M. Ngai J. Scott K. (2010). The molecular basis for water taste in *Drosophila*. *Nature* 465 91–95. 10.1038/nature09011 20364123 PMC2865571

[B8] Chen Y. D. Dahanukar A. (2017). Molecular and cellular organization of taste neurons in adult *Drosophila* pharynx. *Cell Rep.* 21 2978–2991. 10.1016/j.celrep.2017.11.041 29212040 PMC5736000

[B9] Chen Z. Wang Q. Wang Z. (2010). The amiloride-sensitive epithelial Na + channel PPK28 is essential for drosophila gustatory water reception. *J. Neurosci.* 30 6247–6252. 10.1523/JNEUROSCI.0627-10.2010 20445050 PMC6632709

[B10] Chowdhury Z. S. Sato K. Yamamoto D. (2017). The core-promoter factor TRF2 mediates a fruitless action to masculinize neurobehavioral traits in *Drosophila*. *Nat. Commun.* 8:1480. 10.1038/s41467-017-01623-z 29133872 PMC5684138

[B11] Clowney E. J. Iguchi S. Bussell J. J. Scheer E. Ruta V. (2015). Multimodal chemosensory circuits controlling male courtship in *Drosophila*. *Neuron* 87 1036–1049. 10.1016/j.neuron.2015.07.025 26279475 PMC4560615

[B12] Clyne P. J. Warr C. G. Carlson J. R. (2000). Candidate taste receptors in *Drosophila*. *Science* 287 1830–1834. 10.1126/science.287.5459.1830 10710312

[B13] Dambly-Chaudiere C. Jamet E. Burri M. Bopp D. Basler K. Hafen E. (1992). The paired box gene pox neuro: a determinant of poly-innervated sense organs in *Drosophila*. *Cell* 69 159–172. 10.1016/0092-8674(92)90127-X 1348214

[B14] Deshpande S. A. Carvalho G. B. Amador A. Phillips A. M. Hoxha S. Lizotte K. J. (2014). Quantifying *Drosophila* food intake: comparative analysis of current methodology. *Nat. Methods* 11 535–540. 10.1038/nmeth.2899 24681694 PMC4008671

[B15] Deshpande S. A. Yamada R. Mak C. M. Hunter B. Soto Obando A. Hoxha S. (2015). Acidic food Ph increases palatability and consumption and extends *Drosophila* lifespan. *J. Nutr.* 145 2789–2796. 10.3945/jn.115.222380 26491123 PMC4656910

[B16] Dethier V. G. (1976). *The Hungry Fly :A Physiological Study of the Behavior Associated with Feeding.* Cambridge, MA: Harvard University Press.

[B17] Devineni A. V. Heberlein U. (2009). Preferential ethanol consumption in *Drosophila* models features of addiction. *Curr. Biol.* 19 2126–2132. 10.1016/j.cub.2009.10.070 20005106 PMC2805771

[B18] Diaper D. C. Adachi Y. Sutcliffe B. Humphrey D. M. Elliott C. J. Stepto A. (2013). Loss and gain of *Drosophila* TDP-43 impair synaptic efficacy and motor control leading to age-related neurodegeneration by loss-of-function phenotypes. *Hum. Mol. Genet.* 22 1539–1557. 10.1093/hmg/ddt005 23307927 PMC3605831

[B19] Dus M. Ai M. Suh G. S. (2013). Taste-independent nutrient selection is mediated by a brain-specific Na + /solute co-transporter in *Drosophila*. *Nat. Neurosci.* 16 526–528. 10.1038/nn.3372 23542692 PMC3637869

[B20] Dus M. Lai J. S. Gunapala K. M. Min S. Tayler T. D. Hergarden A. C. (2015). Nutrient Sensor in the brain directs the action of the brain-gut axis in *Drosophila*. *Neuron* 87 139–151. 10.1016/j.neuron.2015.05.032 26074004 PMC4697866

[B21] Dus M. Min S. Keene A. C. Lee G. Y. Suh G. S. (2011). Taste-independent detection of the caloric content of sugar in *Drosophila*. *Proc. Natl. Acad. Sci. U.S.A.* 108 11644–11649. 10.1073/pnas.1017096108 21709242 PMC3136275

[B22] Falk R. Bleiseravivi N. Atidia J. (1976). Labellar taste organs of *Drosophila melanogaster*. *J. Morphol.* 150 327–341. 10.1002/jmor.1051500206 30261703

[B23] Freeman E. G. Dahanukar A. (2015). Molecular neurobiology of *Drosophila* taste. *Curr. Opin. Neurobiol.* 34 140–148. 10.1016/j.conb.2015.06.001 26102453 PMC4577450

[B24] Galindo K. Smith D. P. (2001). A large family of divergent *Drosophila* odorant-binding proteins expressed in gustatory and olfactory sensilla. *Genetics* 159 1059–1072. 11729153 10.1093/genetics/159.3.1059 PMC1461854

[B25] Gendre N. Luer K. Friche S. Grillenzoni N. Ramaekers A. Technau G. M. (2004). Integration of complex larval chemosensory organs into the adult nervous system of *Drosophila*. *Development* 131 83–92. 10.1242/dev.00879 14645122

[B26] Gendron C. M. Kuo T. H. Harvanek Z. M. Chung B. Y. Yew J. Y. Dierick H. A. (2014). *Drosophila* life span and physiology are modulated by sexual perception and reward. *Science* 343 544–548. 10.1126/science.1243339 24292624 PMC4042187

[B27] Ghysen A. Dambly-Chaudiere C. (2000). A genetic programme for neuronal connectivity. *Trends Genet.* 16 221–226. 10.1016/S0168-9525(99)01969-1 10782116

[B28] Houot B. Gigot V. Robichon A. Ferveur J. F. (2017). Free flight odor tracking in *Drosophila*: effect of wing chemosensors, sex and pheromonal gene regulation. *Sci. Rep.* 7:40221. 10.1038/srep40221 28067325 PMC5220339

[B29] Hussain A. Zhang M. Ucpunar H. K. Svensson T. Quillery E. Gompel N. (2016). Ionotropic chemosensory receptors mediate the taste and smell of polyamines. *PLoS Biol.* 14:e1002454. 10.1371/journal.pbio.1002454 27145030 PMC4856413

[B30] Jeong Y. T. Shim J. Oh S. R. Yoon H. I. Kim C. H. Moon S. J. (2013). An odorant-binding protein required for suppression of sweet taste by bitter chemicals. *Neuron* 79 725–737. 10.1016/j.neuron.2013.06.025 23972598 PMC3753695

[B31] Joseph R. M. Devineni A. V. King I. F. Heberlein U. (2009). Oviposition preference for and positional avoidance of acetic acid provide a model for competing behavioral drives in *Drosophila*. *Proc. Natl. Acad. Sci. U.S.A.* 106 11352–11357. 10.1073/pnas.0901419106 19541615 PMC2698888

[B32] Joseph R. M. Heberlein U. (2012). Tissue-specific activation of a single gustatory receptor produces opposing behavioral responses in *Drosophila*. *Genetics* 192 521–532. 10.1534/genetics.112.142455 22798487 PMC3454881

[B33] Joseph R. M. Sun J. S. Tam E. Carlson J. R. (2017). A receptor and neuron that activate a circuit limiting sucrose consumption. *eLife* 6:e24992. 10.7554/eLife.24992 28332980 PMC5388533

[B34] Kang K. Pulver S. R. Panzano V. C. Chang E. C. Griffith L. C. Theobald D. L. (2010). Analysis of *Drosophila* TRPA1 reveals an ancient origin for human chemical nociception. *Nature* 464 597–600. 10.1038/nature08848 20237474 PMC2845738

[B35] Kim H. Jeong Y. T. Choi M. S. Choi J. Moon S. J. Kwon J. Y. (2017). Involvement of a Gr2a-expressing *Drosophila* pharyngeal gustatory receptor neuron in regulation of aversion to high-salt foods. *Mol. Cells* 40 331–338. 10.14348/molcells.2017.0028 28535667 PMC5463041

[B36] Koganezawa M. Shimada I. (2002). Novel odorant-binding proteins expressed in the taste tissue of the fly. *Chem. Senses* 27 319–332. 10.1093/chemse/27.4.319 12006372

[B37] Koh T. W. He Z. Gorur-Shandilya S. Menuz K. Larter N. K. Stewart S. (2014). The *Drosophila* IR20a clade of ionotropic receptors are candidate taste and pheromone receptors. *Neuron* 83 850–865. 10.1016/j.neuron.2014.07.012 25123314 PMC4141888

[B38] Kojima T. Furuyama A. Isono K. Hamada T. Ohsuga K. Takada S. (2018). Effects of salt taste disorder on behavior and lifespan in *Drosophila melanogaster*. *J. Oral Biosci.* 60 15–20. 10.1016/j.job.2018.01.001

[B39] Krstic D. Boll W. Noll M. (2009). Sensory integration regulating male courtship behavior in *Drosophila*. *PLoS One* 4:e4457. 10.1371/journal.pone.0004457 19214231 PMC2636894

[B40] LeDue E. E. Chen Y. C. Jung A. Y. Dahanukar A. Gordon M. D. (2015). Pharyngeal sense organs drive robust sugar consumption in *Drosophila*. *Nat. Commun.* 6:6667. 10.1038/ncomms7667 25807033 PMC4375776

[B41] Liman E. R. Zhang Y. V. Montell C. (2014). Peripheral coding of taste. *Neuron* 81 984–1000. 10.1016/j.neuron.2014.02.022 24607224 PMC3994536

[B42] Lin C. C. Prokop-Prigge K. A. Preti G. Potter C. J. (2015). Food odors trigger *Drosophila* males to deposit a pheromone that guides aggregation and female oviposition decisions. *eLife* 4:e08688. 10.7554/eLife.08688 26422512 PMC4621432

[B43] Liu C. Bai X. B. Sun J. H. Zhang X. F. Li Y. (2015). Behavioral switch of food preference upon sugar deficiency is regulated by GPCRs in *Drosophila*. *J. Genet. Genom.* 42 409–412. 10.1016/j.jgg.2015.05.004 26233896

[B44] Liu T. Starostina E. Vijayan V. Pikielny C. W. (2012). Two *Drosophila* DEG/ENaC channel subunits have distinct functions in gustatory neurons that activate male courtship. *J. Neurosci.* 32 11879–11889. 10.1523/JNEUROSCI.1376-12.2012 22915128 PMC3460529

[B45] Lu B. Lamora A. Sun Y. Welsh M. J. Ben-Shahar Y. (2012). ppk23-Dependent chemosensory functions contribute to courtship behavior in *Drosophila melanogaster*. *PLoS Genet.* 8:e1002587. 10.1371/journal.pgen.1002587 22438833 PMC3305452

[B46] Lu B. Zelle K. M. Seltzer R. Hefetz A. Ben-Shahar Y. (2014). Feminization of pheromone-sensing neurons affects mating decisions in *Drosophila males*. *Biol. Open* 3 152–160. 10.1242/bio.20147369 24463366 PMC3925318

[B47] Masek P. Keene A. C. (2013). *Drosophila* fatty acid taste signals through the PLC pathway in sugar-sensing neurons. *PLoS Genet.* 9:e1003710. 10.1371/journal.pgen.1003710 24068941 PMC3772025

[B48] Mellert D. J. Knapp J. M. Manoli D. S. Meissner G. W. Baker B. S. (2010). Midline crossing by gustatory receptor neuron axons is regulated by fruitless, doublesex and the Roundabout receptors. *Development* 137 323–332. 10.1242/dev.045047 20040498 PMC2799163

[B49] Minocha S. Boll W. Noll M. (2017). Crucial roles of pox neuro in the developing ellipsoid body and antennal lobes of the *Drosophila* brain. *PLoS One* 12:e0176002. 10.1371/journal.pone.0176002 28441464 PMC5404782

[B50] Mitri C. Soustelle L. Framery B. Bockaert J. Parmentier M. L. Grau Y. (2009). Plant insecticide L-canavanine repels *Drosophila* via the insect orphan GPCR DmX. *PLoS Biol.* 7:e1000147. 10.1371/journal.pbio.1000147 19564899 PMC2695807

[B51] Moon S. J. Lee Y. Jiao Y. Montell C. (2009). A *Drosophila* gustatory receptor essential for aversive taste and inhibiting male-to-male courtship. *Curr. Biol.* 19 1623–1627. 10.1016/j.cub.2009.07.061 19765987 PMC2762023

[B52] Murata S. Brockmann A. Tanimura T. (2017). Pharyngeal stimulation with sugar triggers local searching behavior in *Drosophila*. *J. Exp. Biol.* 220 3231–3237. 10.1242/jeb.161646 28684466

[B53] Park J. Lee J. Shim J. Han W. Lee J. Bae Y. C. (2013). dTULP, the *Drosophila melanogaster* homolog of tubby, regulates transient receptor potential channel localization in cilia. *PLoS Genet.* 9:e1003814. 10.1371/journal.pgen.1003814 24068974 PMC3778012

[B54] Raad H. Ferveur J. F. Ledger N. Capovilla M. Robichon A. (2016). Functional gustatory role of chemoreceptors in *Drosophila* wings. *Cell Rep.* 15 1442–1454. 10.1016/j.celrep.2016.04.040 27160896

[B55] Sanchez-Alcaniz J. A. Zappia G. Marion-Poll F. Benton R. (2017). A mechanosensory receptor required for food texture detection in *Drosophila*. *Nat. Commun.* 8:14192. 10.1038/ncomms14192 28128210 PMC5290141

[B56] Schneider J. Dickinson M. H. Levine J. D. (2012). Social structures depend on innate determinants and chemosensory processing in *Drosophila*. *Proc. Natl. Acad. Sci. U.S.A.* 109(Suppl. 2), 17174–17179. 10.1073/pnas.1121252109 22802679 PMC3477376

[B57] Schneider J. Levine J. D. (2014). Automated identification of social interaction criteria in *Drosophila melanogaster*. *Biol. Lett.* 10:20140749. 10.1098/rsbl.2014.0749 25354920 PMC4272216

[B58] Scott K. (2018). Gustatory processing in *Drosophila melanogaster*. *Annu. Rev. Entomol.* 63 15–30. 10.1146/annurev-ento-020117-043331 29324046

[B59] Scott K. Brady R. Jr. Cravchik A. Morozov P. Rzhetsky A. Zuker C. (2001). A chemosensory gene family encoding candidate gustatory and olfactory receptors in *Drosophila*. *Cell* 104 661–673. 10.1016/S0092-8674(01)00263-X 11257221

[B60] Shiraiwa T. Carlson J. R. (2007). Proboscis extension response (PER) assay in *Drosophila*. *J. Vis. Exp.* 3:e193. 10.3791/193 18978998 PMC2535836

[B61] Soldano A. Alpizar Y. A. Boonen B. Franco L. Lopez-Requena A. Liu G. (2016). Gustatory-mediated avoidance of bacterial lipopolysaccharides via TRPA1 activation in *Drosophila*. *eLife* 5:e13133. 10.7554/eLife.13133 27296646 PMC4907694

[B62] Sovik E. Lamora A. Seehra G. Barron A. B. Duncan J. G. Ben-Shahar Y. (2017). *Drosophila* divalent metal ion transporter Malvolio is required in dopaminergic neurons for feeding decisions. *Genes Brain Behav.* 16 506–514. 10.1111/gbb.12375 28220999 PMC5457331

[B63] Starostina E. Liu T. Vijayan V. Zheng Z. Siwicki K. K. Pikielny C. W. (2012). A *Drosophila* DEG/ENaC subunit functions specifically in gustatory neurons required for male courtship behavior. *J. Neurosci.* 32 4665–4674. 10.1523/JNEUROSCI.6178-11.2012 22457513 PMC3324785

[B64] Steck K. Walker S. J. Itskov P. M. Baltazar C. Moreira J. M. Ribeiro C. (2018). Internal amino acid state modulates yeast taste neurons to support protein homeostasis in *Drosophila*. *eLife* 7:e31625. 10.7554/eLife.31625 29393045 PMC5812714

[B65] Stocker R. F. (1994). The organization of the chemosensory system in *Drosophila melanogaster*: a review. *Cell Tissue Res.* 275 3–26. 10.1007/BF00305372 8118845

[B66] Sun F. Wang Y. Zhou Y. Van Swinderen B. Gong Z. Liu L. (2014). Identification of neurons responsible for feeding behavior in the *Drosophila* brain. *Sci. Chin. Life Sci.* 57 391–402. 10.1007/s11427-014-4641-2 24744088

[B67] Ueno K. Ohta M. Morita H. Mikuni Y. Nakajima S. Yamamoto K. (2001). Trehalose sensitivity in *Drosophila* correlates with mutations in and expression of the gustatory receptor gene Gr5a. *Curr. Biol.* 11 1451–1455. 10.1016/S0960-9822(01)00450-X 11566105

[B68] Usui-Aoki K. Matsumoto K. Koganezawa M. Kohatsu S. Isono K. Matsubayashi H. (2005). Targeted expression of Ip3 sponge and Ip3 dsRNA impaires sugar taste sensation in *Drosophila*. *J. Neurogenet.* 19 123–141. 10.1080/01677060600569713 16540404

[B69] Verschut T. A. Carlsson M. A. Anderson P. Hamback P. A. (2017). Sensory mutations in *Drosophila melanogaster* influence associational effects between resources during oviposition. *Sci. Rep.* 7:9352. 10.1038/s41598-017-09728-7 28839208 PMC5570953

[B70] Vijayan V. Thistle R. Liu T. Starostina E. Pikielny C. W. (2014). *Drosophila* pheromone-sensing neurons expressing the ppk25 ion channel subunit stimulate male courtship and female receptivity. *PLoS Genet.* 10:e1004238. 10.1371/journal.pgen.1004238 24675786 PMC3967927

[B71] Wang K. Y. Guo Y. M. Wang F. Wang Z. R. (2011). *Drosophila* TRPA channel painless inhibits male-male courtship behavior through modulating olfactory sensation. *PLoS One* 6:e25890. 10.1371/journal.pone.0025890 22073144 PMC3206795

[B72] Weiss L. A. Dahanukar A. Kwon J. Y. Banerjee D. Carlson J. R. (2011). The molecular and cellular basis of bitter taste in Drosophila. *Neuron* 69 258–272. 10.1016/j.neuron.2011.01.001 21262465 PMC3033050

[B73] Yanagawa A. Chabaud M. A. Imai T. Marion-Poll F. (2018). Olfactory cues play a significant role in removing fungus from the body surface of *Drosophila melanogaster*. *J. Invertebr. Pathol.* 151 144–150. 10.1016/j.jip.2017.11.011 29175531

[B74] Yanagawa A. Guigue A. M. Marion-Poll F. (2014). Hygienic grooming is induced by contact chemicals in *Drosophila melanogaster*. *Front. Behav. Neurosci.* 8:254. 10.3389/fnbeh.2014.00254 25100963 PMC4107972

[B75] Yang Z. Yu Y. Zhang V. Tian Y. Qi W. Wang L. (2015). Octopamine mediates starvation-induced hyperactivity in adult *Drosophila*. *Proc. Natl. Acad. Sci. U.S.A.* 112 5219–5224. 10.1073/pnas.1417838112 25848004 PMC4413307

[B76] Yilmazer Y. B. Koganezawa M. Sato K. Xu J. Yamamoto D. (2016). Serotonergic neuronal death and concomitant serotonin deficiency curb copulation ability of *Drosophila* platonic mutants. *Nat. Commun.* 7:13792. 10.1038/ncomms13792 27958269 PMC5159827

[B77] Zhang Y. V. Aikin T. J. Li Z. Montell C. (2016). The basis of food texture sensation in *Drosophila*. *Neuron* 91 863–877. 10.1016/j.neuron.2016.07.013 27478019 PMC4990472

[B78] Zhang Y. V. Ni J. Montell C. (2013). The molecular basis for attractive salt-taste coding in *Drosophila*. *Science* 340 1334–1338. 10.1126/science.1234133 23766326 PMC4091975

